# Factors Associated With Renal Involvement in Primary Sjögren's Syndrome: A Meta-Analysis

**DOI:** 10.3389/fmed.2020.614482

**Published:** 2020-11-26

**Authors:** Ruping Hong, Dong Xu, Evelyn Hsieh, Yirong Xiang, Jiuliang Zhao, Qian Wang, Xinping Tian, Mengtao Li, Yan Zhao, Xiaofeng Zeng

**Affiliations:** ^1^Department of Rheumatology, Peking Union Medical College Hospital, Peking Union Medical College & Chinese Academy of Medical Science, National Clinical Research Center for Dermatologic and Immunologic Diseases (NCRC-DID), Key Laboratory of Rheumatology and Clinical Immunology, Ministry of Education, Beijing, China; ^2^Section of Rheumatology, Allergy and Immunology, Yale University School of Medicine, New Haven, CT, United States

**Keywords:** Sjögren's syndrome, renal, kidney, arthralgia, anti-SSB antibody

## Abstract

**Background:** Renal impairment is a critical complication in primary Sjögren's syndrome (pSS), resulting in chronic renal disease and even death. This meta-analysis was designed to find out the relevant factors of renal involvement in pSS.

**Methods:** PubMed, EMBASE, Cochrane Library, Scopus, and Web of Science were systemically searched until August 30, 2019. Studies were selected according to inclusion criteria, and data was extracted by two researchers independently. The Newcastle-Ottawa Scale was applied for quality assessment. Random- and fixed-effects models were used in this meta-analysis based on the result of the heterogeneity test. Meanwhile, a sensitivity analysis was conducted to investigate the cause of heterogeneity. Publication bias was shown in the funnel plot and evaluated further by Begg's and Egger's tests.

**Results:** Of the 9,989 articles identified, five articles enrolling 1,867 pSS patients were included in the final analysis, 533 with and 1,334 without renal involvement. There was no statistical significance in age and gender between these two groups. According to the meta-analysis, anti-SSB antibody, and arthralgia showed a significant association with renal involvement in pSS, the overall odds ratio (OR) values of which were 1.51 (95% CI, 1.16–1.95) and 0.59 (95% CI, 0.46–0.74), respectively. On the other hand, the overall OR values of anti-SSA antibody, rheumatoid factor, dry eyes, and labial salivary gland biopsy were just 0.90 (95% CI, 0.49–1.64), 1.05 (95% CI, 0.59–1.86), 0.60 (95% CI, 0.34–1.06), and 1.38 (95% CI, 0.98–1.95), respectively.

**Conclusion:** The presence of anti-SSB antibody is positively associated with renal involvement in pSS, while arthralgia is inversely associated. Large-scale prospective cohort studies are needed in the future to identify further risk factors.

## Introduction

Primary Sjögren's syndrome (pSS) is a chronic autoimmune disease characterized by the involvement of exocrine glands, usually resulting in xerostomia and xeropthalmia (1), and it is also the second common autoimmune disease in middle-aged women (2). Additionally, it has been realized that pSS could have an extensive effect on many organs in the human body, among which the kidney is an important target (3). The exact prevalence of renal involvement in pSS remains unknown, considering the difference in diagnosis criteria of SS, study populations, and the definition of renal impairment. According to studies by far, it ranges from 0.3 to 33.5% (4–9). The nephropathy of pSS could be further divided into two categories: tubulointerstitial nephritis (TIN) and, less frequently, glomerular involvement (10). The presentation of TIN could be insidious without clinically significant signs or symptoms, so there is a possibility that these patients would develop terminal chronic kidney disease due to not receiving proper treatment, even though this kind of nephropathy could be alleviated after treatment in many studies (4, 11, 12). Meanwhile, glomerular involvement is usually accompanied by overt symptoms such as hypertension, evident proteinuria, and hematuria (13), which could be treated earlier after differential diagnosis. A study by Goules et al. revealed that pSS patients with glomerulonephritis showed increased mortality but a lower proportion of chronic renal failure compared with those with interstitial nephritis (8).

Therefore, we designed this meta-analysis to find out clinical factors associated with renal involvement in pSS. Based on that, for those patients with specific clinical factors, clinicians are more likely to pay attention to renal impairment and then give them appropriate treatment earlier.

## Methods

This meta-analysis was conducted according to the instruction of the Preferred Reporting Items for Systematic Reviews and Meta-Analyses (PRISMA).

### Literature Search

We searched PubMed (from 1948 to August 2019), EMBASE (from 1980 to August 2019), Cochrane Library (from 2000 to August 2019), Scopus (from 1960 to August 2019), and Web of Science (from 1950 to August 2019) to identify all relevant articles. These following keywords or MeSH terms were used: “Sjogren's syndrome,” “Sjogren^*^ syndrome,” “sicca syndrome,” “SS,” “kidney,” and “renal.” There was no addition of any filter such as language, species, or article types during the search process. Take the research strategy in PubMed as an example:

#1, “Search Sjogren's syndrome [MeSH Terms]”, 12,329.
#2, “Search (sjogren^*^ syndrome [Title/Abstract] OR sicca syndrome [Title/Abstract]),” 15,785.
#3, “Search (#1 OR #2),” 17,578.
#4, “Search (kidney [MeSH Terms]) OR (renal [Title/Abstract] OR kidney^*^[Title/Abstract]),” 935,287.
#5, “Search (#3 AND #4),” 1,076.

### Study Selection

We imported all articles into EndNote software (version X9) for further screening. The inclusion criteria were as follows: (1) all enrolled patients in the study with a diagnosis of pSS fulfilling the European criteria or the 2002 classification criteria of the American-European Consensus Group (AECG); (2) comparing pSS patients with renal involvement and those without in terms of clinical and laboratory characteristics; and (3) studies published in English. Two researchers assessed study eligibility and completed the study selection, respectively. These two researchers would discuss any discrepancies together, and a consensus was finally reached.

### Data Collection

Data was collected independently by two researchers. The following items were extracted from each study included: country, study type, time span, diagnosis of SS, the definition of renal involvement, clinical presentations of renal involvement, pathological results of renal biopsy, the mean age of participants, gender, and comparative items in the study. Discrepancies were discussed and solved by the researchers.

### Quality Assessment

The Newcastle-Ottawa Scale (http://www.ohri.ca/programs/clinical_epidemiology/oxford.asp) was used to assess the quality of the studies, in which each study was scored based on the quality of selection, comparability, and exposure. The quality of selection was further divided into four aspects including the definition of the case, the representativeness of the cases, the selection of controls, and the definition of controls. The quality of exposure involved its ascertainment, the same method of ascertainment for case and controls, and non-response rate. In total, there were eight items in this scale with a score up to nine. Two researchers completed quality assessment independently, and a third one was needed to solve any discrepancies if necessary.

### Statistical Analysis

Items were included for meta-analysis if they met the following criteria: (1) analyzed in at least three articles; (2) presented in the same form; and (3) the statistical significance of which not in consensus among different articles. As for those items with significant heterogeneity test (*P* < 0.05), the random-effects model was adopted in forest plots, or else, the fixed-effects model was used. Meanwhile, a sensitivity analysis was conducted by leaving out each included study one by one to investigate heterogeneity further. In addition, publication bias was evaluated by funnel plots and Begg's and Egger's tests. Data analyses were conducted using Stata/SE 15.0 and Review Manager 5.3 software, and any outcome, with a two-tailed *P* < 0.05, was considered significant.

## Results

### Literature Search

**Figure 1:**
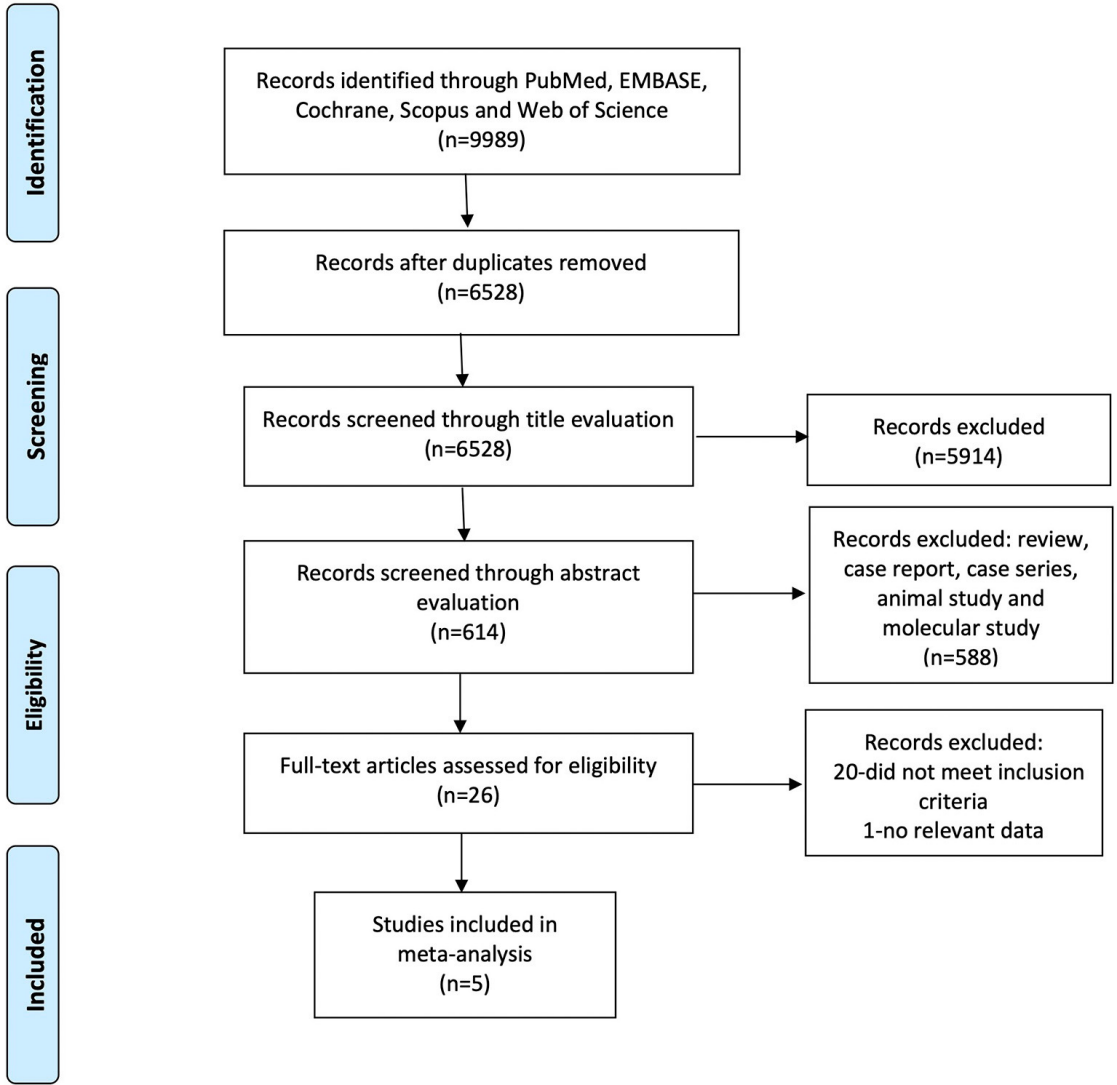
Flowchart of screening studies in this meta-analysis.

A total of 9,989 articles were found by a comprehensive search in PubMed, EMBASE, Cochrane Library, Scopus, and Web of Science, of which 6,528 were left after the removal of duplicates. We excluded 5,914 articles unrelated to pSS-associated renal involvement after title evaluation. Among the remaining 614 articles, we screened the abstract further. Case report, case series, reviews, and molecular study were excluded. Finally, we read the full text of 26 articles for eligibility. Those studies not providing clinical data needed were excluded. Meanwhile, owing to the paucity of relevant articles or data, we did not conduct subgroup analysis considering the different subtypes of renal involvement such as TIN and glomerulonephritis. We finally included five articles for further data collection and analysis. Figure 1 shows the flowchart of article screening in this meta-analysis.

### Basic Characteristics of Included Studies

Table 1 summarizes the five included studies' essential characteristics, which were carried out in China, Italy, and India. Bossini et al. used the European criteria for SS diagnosis, while the 2002 AECG criteria were applied in the other four studies. As for the definition of renal involvement, Yang et al. only chose biopsy-proven cases, the indications of which were nephrotic range proteinuria, acute nephritic syndrome, and unexplained renal insufficiency. On the other hand, the other four studies took an overall consideration of urine analysis, serological analysis, and imaging evaluation including routine urine test, urine protein estimation, urine pH, serum creatine, serum electrolytes, and arterial blood gases. Additionally, renal function tests were performed when necessary, and kidney biopsy was applied as appropriate. Finally, 1,867 pSS patients were enrolled in these studies, 533 with and 1,334 without renal involvement.

**Table 1:** Baseline characteristics of the included studies.

**Items**	**Bossini et al. (6)**	**Yang et al. (14)**	**Jain et al. (15)^#^**	**Luo et al. (16)**	Luo et al. (17)^*^
Country	Italy	China	India	China	China
Study type	Prospective	Retrospective	Prospective	Cross-sectional	Cross-sectional
Time span	NR	2005–2015	2015–2016	2013–2017	2013–2017
Diagnosis criteria of SS	European criteria	2002 AECG criteria	2002 AECG criteria	2002 AECG criteria	2002 AECG criteria
**Patients**					
pSS-renal, *N*	16	103	35	217	162
Female, *N* (%)	16 (100.00)	91 (88.35)	35 (100.00)	192 (88.48)	150 (92.59)
Mean age, years	53	45.2	37.6	58.4	49.9
pSS-only, *N*	36	206	35	217	840
Female, *N* (%)	33 (91.67)	186 (90.29)	33 (94.28)	192 (88.48)	779 (92.74)
Mean age, years	55	43.3	40.6	57.3	49.5
**Clinical presentations of renal involvement**					
CCR, ml/min	75.43 ± 20.40	NR	71.85 ± 18.04	62.88 ± 33.28	NR
RTA, *N*	3	58	29	64	12
Type I, *N*	3	NR	29	60	NR
Type II, *N*	0	NR	0	4	NR
Hypokalemia, *N*	4	46	22	68	NR
Proteinuria, *N*	12	53	NR	116	NR
Hematuria, *N*	5	41	NR	64	1
**Pathological types of renal biopsy**					
*N*	9	103	17	30	12
TIN, *N*	6	53	9	5	6
GN, *N*	3	50	0	21	5
TIN+GN, *N*	0	0	4	4	0

*NR, not reported; pSS, primary Sjögren's syndrome; AECG, American-European Consensus Group; CCR, creatinine clearance rate; RTA, renal tubular acidosis; TIN, tubulointerstitial nephritis; GN, glomerulonephritis*.

# *Renal biopsy was normal in four patients of which one had pyuria and one had renal dysfunction. All had type I RTA*.

* *Renal biopsy showed renal impairment owing to diabetes mellitus in one patient*.

Among these, the creatinine clearance rate was lower in patients with renal involvement. Of the five studies, renal tubular acidosis (RTA) was a critical clinical presentation, and type I RTA had a predominance, which was usually accompanied by hypokalemia. Proteinuria and hematuria were not rare in these studies. When it came to renal biopsy, according to Table 1, the result showed an inclination for TIN, even though, in the study of “Luo, 2018,” glomerulonephritis dominated (21/30). The researcher ascribed this phenomenon to the higher proportion of patients with proteinuria, which was more likely to indicate glomerular lesion and be indicative of renal biopsy. There was no statistical significance in age and gender observed in these studies. Quality assessment using the Newcastle-Ottawa Scale is illustrated in Table 2.

**Table 2:** Quality assessment of each included study.

	**Bossini et al. (6)**	**Yang et al. (14)**	**Jain et al. (15)**	**Luo et al. (16)**	**Luo et al. (17)**
Selection					
Is the case definition adequate	1	1	1	1	1
Representativeness of the cases	1	0	1	1	1
Selection of controls	0	0	0	0	0
Definition of controls	1	1	1	1	1
Comparability	2	2	2	2	2
Exposure					
Ascertainment of exposure	1	1	1	1	1
Same method of ascertainment for case and controls	1	1	1	1	1
Non-response rate	1	1	1	1	1
Total score	8	7	8	8	8

### Factors Associated With Renal Involvement

**Figure 2:**
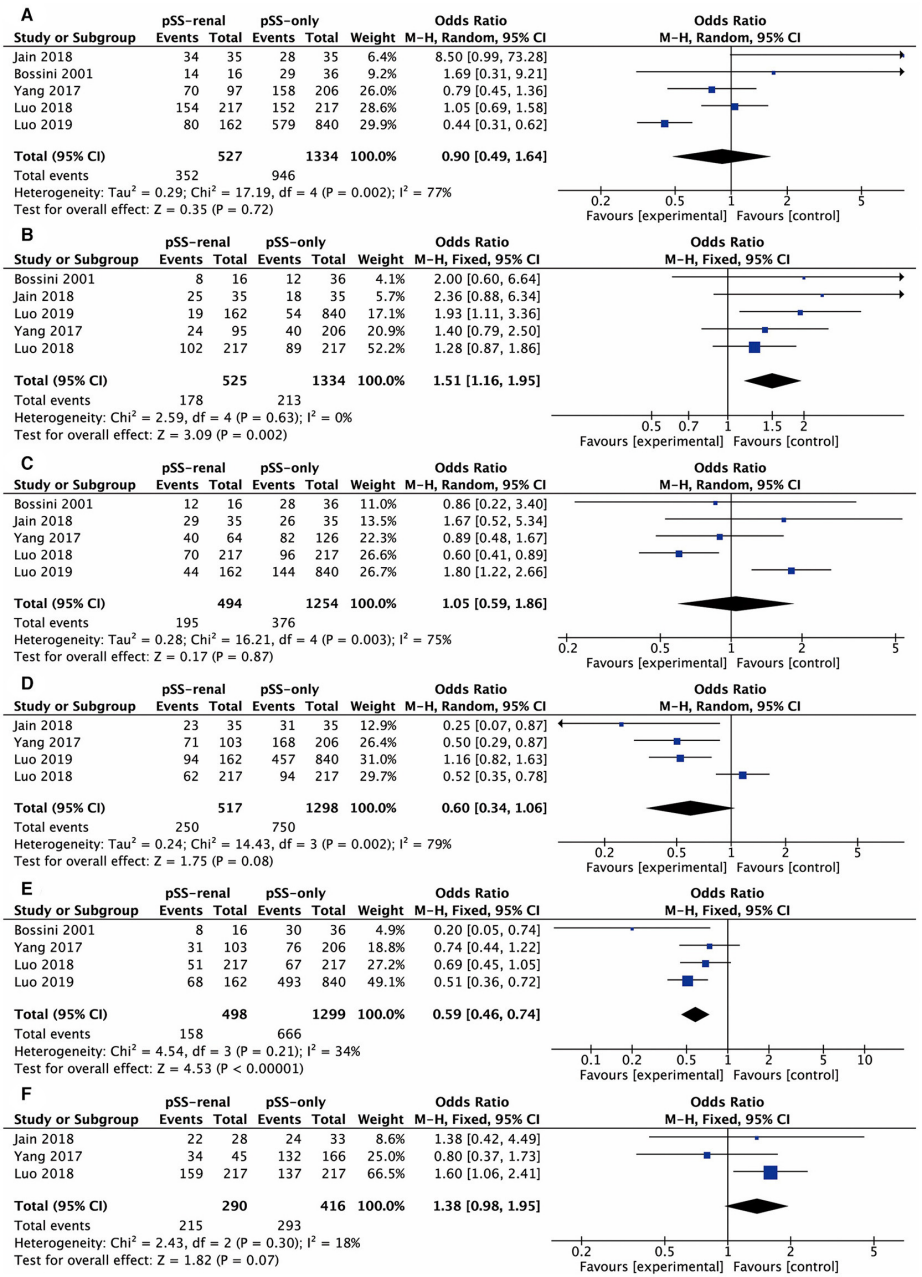
Forest plots of relevant factors of renal involvement in primary Sjögren's syndrome. **(A)** Anti-SSA antibody; **(B)** anti-SSB antibody; **(C)** rheumatoid factor; **(D)** dry eyes; **(E)** arthralgia; **(F)** labial salivary gland biopsy.

These comparative items were analyzed in at least three studies: antinuclear antibody (ANA), anti-SSA antibody, anti-SSB antibody, rheumatoid factor (RF), dry eyes, dry mouth, arthralgia, and labial salivary gland biopsy (LSGB), the results of which were all presented in the form of positive rate. The comparative results of ANA and dry mouth were not significant in all four studies involved. Figure 2 shows the results of the meta-analysis of the remaining six items. The overall odds ratio (OR) values of anti-SSA antibody, anti-SSB antibody, RF, dry eyes, arthralgia, and LSGB were 0.90 (95% CI, 0.49–1.64), 1.51 (95% CI, 1.16–1.95), 1.05 (95% CI, 0.59–1.86), 0.60 (95% CI, 0.34–1.06), 0.59 (95% CI, 0.46–0.74), and 1.38 (95% CI, 0.98–1.95), respectively. We further conducted subgroup analysis considering the different diagnostic criteria of pSS. “Bossini, 2001” was involved with the meta-analysis of anti-SSA antibody, anti-SSB antibody, RF, and arthralgia. There were no significant discrepancies between the whole analysis and subgroup analysis, and the final conclusions remained the same.

### Sensitivity Analysis

Sensitivity analysis suggested that the result of the anti-SSB antibody was stable, and arbitrarily deleting one study would not change its heterogeneity. Nevertheless, “Luo, 2019” had an influence on heterogeneity for anti-SSA antibody (*I*^2^ = 77% vs. *I*^2^ = 40%), RF (*I*^2^ = 75% vs. *I*^2^ = 11%), and dry eyes (*I*^2^ = 79% vs. *I*^2^ = 0%). Also, “Bossini, 2001” was the major cause of heterogeneity for arthralgia (*I*^2^ = 34% vs. *I*^2^ = 0%).

### Publication Bias

**Figure 3:**
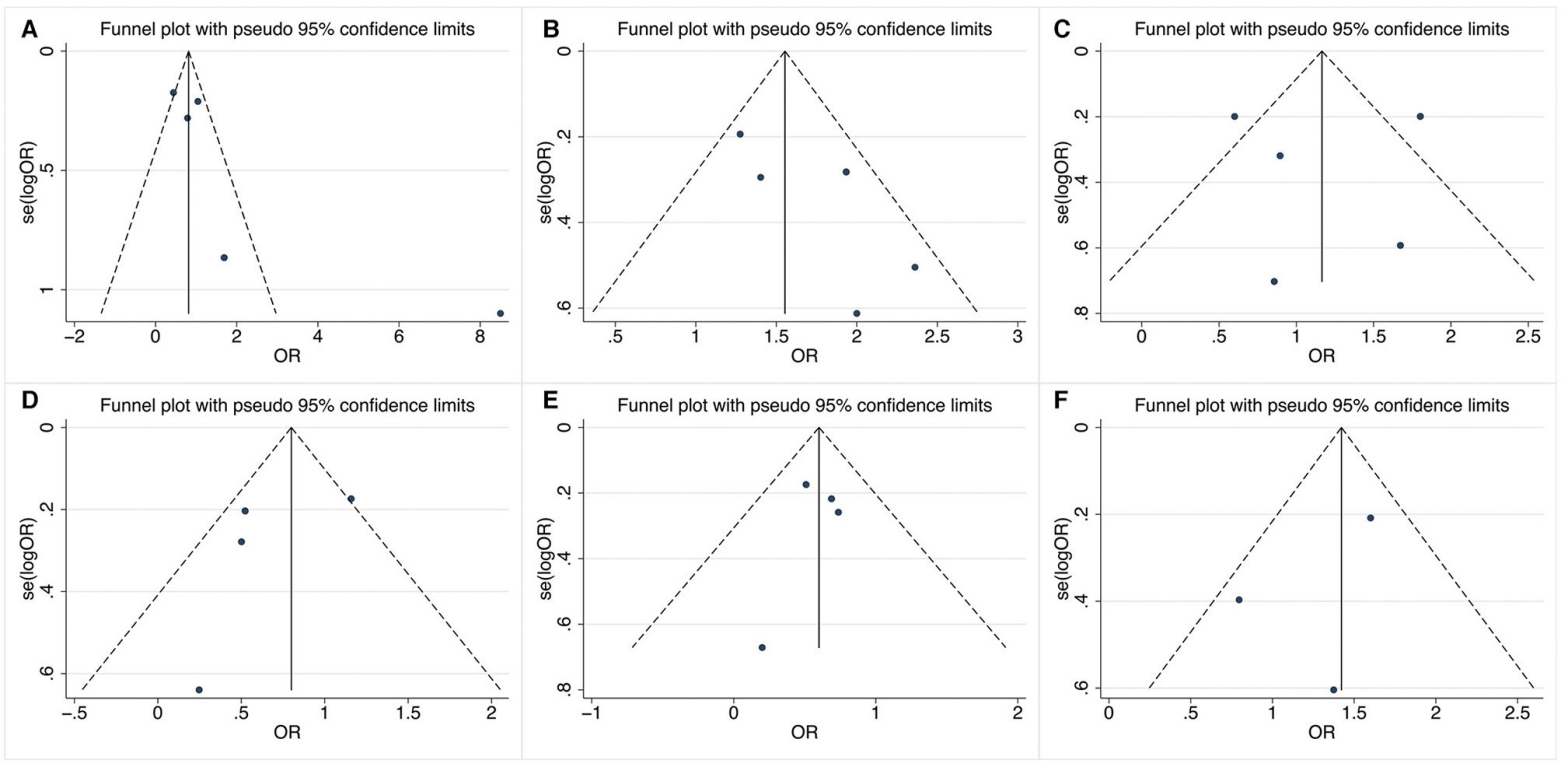
Funnel plots of relevant factors of renal involvement in primary Sjögren's syndrome. **(A)** Anti-SSA antibody; **(B)** anti-SSB antibody; **(C)** rheumatoid factor; **(D)** dry eyes; **(E)** arthralgia; **(F)** labial salivary gland biopsy.

Figure 3 shows the results of funnel plots to assess the possibility of publication bias. Obvious asymmetry of shape was not observed in these funnel plots. In addition, Begg's and Egger's tests did not reveal statistical significance with a *P*-value more than 0.05, indicating no publication bias.

## Discussion

Renal involvement is a common complication in pSS with a variety of clinical presentations, which greatly affects the prognosis of patients. Ramos-Casals et al. revealed that renal failure (defined as creatinine more than 1.3 mg/dl) was found in ~24% of 198 pSS patients with renal involvement (18). Bearing the severity in mind, we conducted this meta-analysis to identify relevant factors in order to manage these patients better.

Among these comparative items, the existence of anti-SSB antibody was positively correlated with renal impairment in pSS. According to previous studies, anti-SSA/SSB antibodies were known to be related to extraglandular manifestations (19). For these pSS patients with RTA, the test of the anti-SSB antibody showed more positive results (3) and the titer of the anti-SSB antibody was higher than that in those without (20). Furthermore, in other autoimmune diseases such as systemic lupus erythematosus (SLE), the positive anti-SSB antibody test was identified to be one of the risk factors for renal recurrence in those with lupus nephritis type IV (21). Meanwhile, it was also observed to have a connection with nodal or spleen enlargement and with an increased level of gamma-globulins in SS, SLE, and progressive systemic sclerosis (22). All of these indicated immunologic disorder participating in the development of nephropathy in pSS. There were many studies showing lymphocytic infiltration as well as autoantibodies to components of renal tubules in TIN (23–26). Cryoglobulinemia and immune-complex deposition were found in glomerulonephritis (8, 27). As for these two types of renal impairment in pSS, the anti-SSB antibody did not show a predominance in any one of them (8, 11, 28, 29). The treatment of abatacept in pSS could reduce activated circulating follicular helper T cell to attenuate B cell hyperactivity, in which the titer of the anti-SSB antibody decreased as well as EULAR Sjogren's syndrome disease activity index (ESSDAI) and Clinical ESSDAI scores (30). However, Jasiek et al. found that the presence of anti-SSA and anti-SSB antibodies was related to no or poor response to the treatment of CSs and/or rituximab in pSS patients with TIN (31). More research is necessary to fully understand the relationship between the anti-SSB antibody and the pathophysiology of nephropathy in pSS.

The anti-SSA antibody was associated with the development or worse prognosis of renal impairment in some studies (31–33). However, we did not observe statistical significance in the anti-SSA antibody. The following reasons may explain this. The anti-SSA antibody was very common in pSS with a positive rate of up to 84.6% (34), while the ratio of renal involvement in pSS was relatively low. The number of patients we enrolled in the present meta-analysis is perhaps not enough to discover the exact association between the anti-SSA antibody and renal disease. Therefore, studies conducted on a larger scale are needed in the future.

pSS patients with renal involvement showed a lower prevalence in arthralgia than those without renal impairment. Articular manifestations are not rare presentations of pSS and could be simultaneous with, precede, or follow the development of sicca syndrome (35). According to previous studies, the prevalence of articular involvement ranges from 20 to 60%, and the most common symptoms are arthralgia and non-erosive polyarthropathy (36, 37). Consistent with this meta-analysis, arthralgia was more frequent in pSS without RTA compared with those with RTA (64 vs. 29%, *P* < 0.05) (38). Similarly, Jain et al. also found fewer articular manifestations in pSS patients with renal involvement (15). However, Fauchais et al. retrospectively analyzed pSS patients with and without articular manifestations, which were defined as arthralgia or non-erosive arthritis involving one or more peripheral joints. They found more renal involvement in pSS patients with articular manifestations than those without (23/188 vs. 11/231, *P* = 0.007) (36). Nevertheless, there was no statistical significance in RTA between these two groups in the study conducted by Pease and co-authors (39). The difference may be explained by the variety of populations as well as the definitions of articular and renal involvement. Considering more articular involvement and extraglandular features correlated with the positive result of RF in pSS (19, 40), it is interesting to explore the association between RF and renal disease, even though it showed no significance in this meta-analysis. In other autoimmune diseases, some studies revealed a negative relationship between RF and renal disease in systemic lupus erythematosus (41, 42).

This meta-analysis did not find an association between renal involvement and dry mouth as well as LSGB. Though the OR value reached 0.60 (95% CI, 0.34–1.06) and 1.38 (95% CI, 0.98–1.95), respectively, they remained insignificant. The pathophysiology of exocrine gland and renal disease in pSS both involved the infiltration of lymphocytes and tissue damage (1). It indicates that different and more specific mechanisms participate in the development of these two complications, respectively.

Inevitably, there are some limitations in this meta-analysis. First, we included five studies into the final analysis, and just three studies were involved with the analysis of LSGB, which would weaken the effect of meta-analysis. Second, owing to the various manifestations of renal impairment and the different detection methods, it hardly unified the definition of renal involvement in these included studies. Third, given that most of them were retrospective studies, we could not reach a causative conclusion. In addition, the authors did not provide enough information of potential confounding factors like medications, which would make our final conclusions a little less convincing and necessary to be strengthened further by well-designed studies in the future. Finally, heterogeneity was an issue always discussed in meta-analysis. The study size and enrollment population may explain the heterogeneity caused by “Bossini, 2001” for arthralgia. Besides, the patient number used in this meta-analysis was the total number of pSS patients with arthralgia or arthritis in “Bossini, 2001,” which would introduce deviation to some extent. “Luo, 2019” was the source of heterogeneity for anti-SSA antibody, RF, and dry eyes. Among these five studies, “Luo, 2019” was the largest one in study size. As for dry eyes, it was a subjective symptom and influenced by many factors such as the way of communication between researchers and patients. In addition, the detection method would impact the result of the anti-SSA antibody, which was different from that in the anti-SSB antibody (43). This also explained why the result of the anti-SSB antibody was stable in the sensitivity analysis.

## Conclusion

This meta-analysis aims to find out the factors associated with renal involvement in pSS. The presence of anti-SSB antibody is positively related to nephropathy in pSS, while arthralgia is inversely related. Large-scale and well-designed prospective cohort studies are necessary in the future to identify risk factors of renal disease in pSS, which is beneficial for the further management of these special individuals.

## Data Availability Statement

The original contributions presented in the study are included in the article/supplementary materials, further inquiries can be directed to the corresponding author/s.

## Author Contributions

ML, YZ, XZ, and EH: conception and design of this study. RH, DX, and YX: literature research and data acquisition. RH, DX, JZ, QW, and XT: data analysis and interpretation. ML, YZ, XZ, and EH: supervision. Each author contributed importantly during manuscript drafting or revision and was accountable for all aspects of the work. The final version to be published was approved by all authors.

## Conflict of Interest

The authors declare that the research was conducted in the absence of any commercial or financial relationships that could be construed as a potential conflict of interest.
